# Cardiorespiratory Fitness and Endothelial Function in Aging Healthy Subjects and Patients With Cardiovascular Disease

**DOI:** 10.3389/fcvm.2022.870847

**Published:** 2022-04-28

**Authors:** Karsten Königstein, Jonathan Wagner, Denis Infanger, Raphael Knaier, Gilles Nève, Christopher Klenk, Justin Carrard, Timo Hinrichs, Arno Schmidt-Trucksäss

**Affiliations:** ^1^Department of Sport, Exercise and Health, Division Sports and Exercise Medicine, University of Basel, Basel, Switzerland; ^2^Clinic for Children and Adolescent Medicine, Städtisches Klinikum Karlsruhe, Karlsruhe, Germany; ^3^Department of Radiology, Ludwig-Maximilians University, Munich, Germany

**Keywords:** aging, cardiovascular disease, primary prevention, secondary prevention, ultrasound, peripheral vascular disease, atherosclerosis

## Abstract

**Background:**

Individuals with a higher lifelong cardiorespiratory fitness show better vascular health with aging. Studies on fitness-related effects on endothelial function either analyzed samples with a narrow age-range or incompletely assessed endothelial responsiveness. This study aims to assess the impact of cardiorespiratory fitness on the association of brachial-arterial flow-mediated vasodilation (FMD) and low flow-mediated vasoconstriction (L-FMC) with age in healthy adults and patients with cardiovascular diseases.

**Methods:**

FMD, L-FMC and $\dot{\text{V}}$O_2_peak were prospectively measured in a population-based sample including 360 healthy adults and 99 patients with cardiovascular disease of European descend. Non-linear models were applied to assess $\dot{\text{V}}$O_2_peak-associated variations in age-related differences of endothelial function independent of classical cardiovascular risk factors.

**Results:**

FMD was negatively associated with age in healthy adults (adjusted R^2^ = 0.27, partial R^2^ = 0.07, *p* < 0.001) and in cardiovascular patients (adjusted R^2^ = 0.29, partial R^2^ = 0.05, *p* = 002). L-FMC showed no association with age. In models predicting the change of FMD with higher age, $\dot{\text{V}}$O_2_peak accounted for 2.8% of variation in FMD (χ^2^(5) = 5.37, *p* = 0.372, *s* = 1.43). Thereby, $\dot{\text{V}}$O_2_peak-stratified changes of FMD started to fan out at around 30 years of age in women and 50 years of age in men, with 7–12% lower values at old age with $\dot{\text{V}}$O_2_peak ≤3rd percentile compared to $\dot{\text{V}}$O_2_peak ≥97th percentile) in both, the healthy sample and in cardiovascular patients.

**Conclusion:**

The statistical effect of cardiorespiratory fitness on the association of FMD with age independent of classical cardiovascular risk factors was small in both, healthy aging adults as well as patients with cardiovascular diseases. Its clinical significance should be assessed further.

## Introduction

Cardiorespiratory fitness is a strong predictor of cardiovascular morbidity and mortality (1, 2). However, less than 50% of the protective effects of regular exercise on the cardiovascular system can be explained by traditional risk factors (3–5). The remaining risk factor gap of more than 50% may, at least partly, be explained by direct effects of exercise-induced hemodynamic changes on functional and structural properties of the vascular walls (6, 7).

This has been demonstrated with brachial arterial flow-mediated vasodilation (FMD), which reflects nitric oxide dependent vasodilator responsiveness of the endothelium toward acute changes in shear stress (8–10). Studies demonstrated that FMD can be improved by exercise, especially when endothelial function is already impaired (11, 12). This seems to be associated to higher peak oxygen consumption ($\dot{\text{V}}$O_2_peak), the gold-standard biomarker of cardiorespiratory fitness (13). However, previous works on samples including individuals of a specific age infrequently report an association between $\dot{\text{V}}$O_2_peak and FMD (14). Especially in young healthy individuals, FMD appears independent of $\dot{\text{V}}$O_2_peak (15). Instead, two studies in confined samples of young and older adults found favorable cross-sectional associations of $\dot{\text{V}}$O_2_peak with low flow-mediated constriction (L-FMC), which reflects the endothelial vasoconstrictor responsiveness toward altered shear stress (16, 17). Therefore, FMD and L-FMC have been supposed as complementary biomarkers of endothelial function (18, 19).

Recently, age-related differences of FMD and L-FMC were demonstrated in a healthy European population sample (20). Some evidence indicates that a lifelong high $\dot{\text{V}}$O_2_peak might effectively support preservation of good endothelial function into old age (13, 15). It was speculated that $\dot{\text{V}}$O_2_peak might modify the association of FMD with age, more so in the elderly compared to young adults (15). Finally, a recent meta-analysis concluded that existing evidence is inconclusive about whether exercise, irrespective of its impact on $\dot{\text{V}}$O_2_peak, is effective to improve FMD in previously sedentary older adults (21). Accordingly, adaptive capacities of the vascular endothelium toward exercise might vary across the life span and depend on the amount of high-intensity exercise. However, direct effects of $\dot{\text{V}}$O_2_peak on the association of endothelial function with age, independent of classical cardiovascular risk factors, have only been inconsistently studied so far, and only endothelial vasodilator function (FMD), but not vasoconstrictor function (L-FMC), has been analyzed.

In addition, no studies involving patients with cardiovascular diseases exist, that include both, FMD and L-FMC. However, FMD and L-FMC are impaired with apparent cardiovascular diseases, such as heart failure (22), coronary artery disease (18) and arterial hypertension (23). In these patients, increased $\dot{\text{V}}$O_2_peak was associated with improved FMD (24, 25) and L-FMC (26), leading to lower rates of morbidity and mortality (27).

A better understanding of the interactions between cardiorespiratory fitness, aging and endothelial function is necessary to improve precision of exercise-based prescriptions in promotion of healthy aging. Therefore, this study analyzed the impact of cardiorespiratory fitness on the interrelation between endothelial function and age in healthy adults and cardiovascular patients. Specifically, it investigated (I) whether age is independently associated with FMD and L-FMC throughout adult lifespan and (II) whether $\dot{\text{V}}$O_2_peak moderates age-related differences of endothelial function. Based on existing evidence (15, 28), the assumption is that age is associated with FMD and L-FMC in a non-linear relationship and that age-related differences of vascular biomarkers depend on the level of $\dot{\text{V}}$O_2_peak.

## Materials and Methods

### Study Design and Participant Characteristics

**FIGURE 1:**
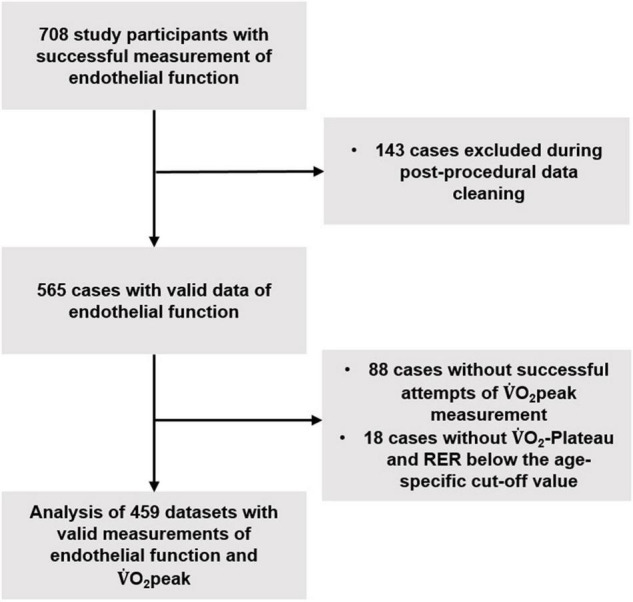
Flow-chart of data acquisition and cleaning. $\dot{\text{V}}$O_2_peak = peak oxygen consumption, RER = respiratory exchange quotient.

This prospective study was approved by the Ethics Committee of Northwestern and Central Switzerland (EKNZ 2017–01451) and conducted between 2018 and 2020 according to the Declaration of Helsinki. Written informed consent was obtained from all study participants before study participation. It was part of the COmPLETE-project (29). A detailed description of the study design is presented elsewhere (29). In summary, the project aimed to analyze age-related differences in physical functioning and to assess the role of physical fitness in this context. For the current study, targeting the interplay between cardiorespiratory fitness and vascular aging, 564 healthy adults between 20 and 91 years of age were recruited based on a randomized and blinded invitation procedure involving rural and urban areas. 144 patients with cardiovascular diseases aged 26 to 91 years stemmed from the vicinity of the cantons Basel-City and Basel-Country. Details about the recruitment procedure can be found elsewhere (29). After post-procedural data cleaning, full datasets of 360 healthy participants and 99 patients with cardiovascular diseases were used for statistical analyses (Figure 1). Empiric and anthropometric data were collected during a medical interview and examination (29).

### Measurement of Endothelial Function

FMD and L-FMC were measured semi-automatically with an ECG-guided high-resolution B-mode ultrasound system (UNEX-EF-38G, UNEX Corp., Nagoya, Japan) following a standardized procedure according to up-to-date methodological recommendations (30). Excellent reliability and precision of this method have been demonstrated before (31). Details of measurement procedure, post-procedural data cleaning and automatic calculation of FMD and L-FMC have been described elsewhere (20). Importantly, lower L-FMC values indicate better vasoconstrictor responsiveness, whereas higher FMD values indicate better vasodilator responsiveness.

### Measurement of Cardiorespiratory Fitness

Cardiopulmonary exercise testing was conducted on an electromagnetically braked cycle ergometer (Ergoselect-200; Ergoline, Bitz, Germany). Gas exchange and ventilatory variables were continuously analyzed (breath-by-breath) using a computer-based system (MetaMax-3B; Cortex Biophysik GmbH, Leipzig, Germany). Before and during tests, participants were encouraged to reach their individual level of maximal exertion (i.e., volitional exhaustion, dyspnea, or fatigue). $\dot{\text{V}}$O_2_peak was defined as the highest 30-second average of $\dot{\text{V}}$O_2_ at any point during the test. Participants without a $\dot{\text{V}}$O_2_-plateau who did not fulfill secondary $\dot{\text{V}}$O_2_peak criteria were excluded from analysis. A complete description of testing procedures, including ramp protocols based on predicted performance can be found elsewhere (32).

### Statistical Analyses

Data analysis was performed using “R” version 3.6.1. Means and standard deviations were calculated for general description of the dataset and the level of significance was set at *p* = 0.05 for all tests. All tests were two-sided. Independent samples *t*-test was used for sex-specific differences between healthy adults and patients with cardiovascular diseases. Linear regression models were used to predict associations of age with FMD and L-FMC. Sample selection accounted for age-related variations of disease burden at different ages, and models were additionally adjusted for sex, mean arterial pressure, heart rate and pre-cuff-inflation diameter, which are major effectors of FMD and L-FMC. All continuous variables were modeled using restricted cubic splines with four knots placed at appropriate quantiles. To evaluate the evidence of a moderating effect of $\dot{\text{V}}$O_2_peak on the relationship between age and FMD, a likelihood ratio test was used comparing a model with an interaction between $\dot{\text{V}}$O_2_peak and age to a model without such an interaction. Model fits were inspected using diagnostic residual plots. The model for FMD exhibited heteroscedastic residuals. Therefore, heteroscedasticity-robust *p*-values and 95%–confidence intervals are presented for this model. To visualize the relationship of FMD with age for different levels of cardiorespiratory fitness, model-predicted change of FMD was plotted for different centiles of $\dot{\text{V}}$O_2_peak (3rd, 15th, 50th, 85th, and 97th) (33) while other variables were fixed at their sex-specific mean. Partial R^2^ was presented to quantify the relative importance of the included variables. To aid interpretation, *p*-values were converted to surprisal values (*s*-values) when appropriate using the formula *s* = −*log*_2_⁡(*p*) (34). The *s*-value provides an intuitive interpretation of the information conveyed by a certain *p*-value against the tested hypothesis. For a specific *s*-value, *p* conveys roughly the same evidence against the tested null hypothesis as seeing all heads in *s* independent tosses of a fair coin.

## Results

### Sample Characteristics

Data of 360 healthy non-smoking adults without cardiovascular disease (48.1% women) and 99 patients with cardiovascular diseases (28.3% women) were analyzed (Figure 1 and Table 1). Physical activity of the subjects was above a population specific average [details provided elsewhere (33)]. Healthy participants had a very low average 10-year risk of cardiovascular disease of 8.2 ± 8.6% (35) and their age- and sex-specific $\dot{\text{V}}$O_2_peak was similar (36) or higher (37, 38) than in other large cohorts of healthy adults. Cardiovascular diseases among the patients were arterial hypertension (*N* = 42), coronary artery disease (*N* = 41), aortic/mitral valve regurgitation (*N* = 5), atrial fibrillation (*N* = 4), dilative cardiomyopathy (*N* = 4), aortic/mitral valve stenosis (*N* = 2) and pulmonary hypertension (*N* = 1). 95 participants received antihypertensive medication including β-blockers in 47 cases. Lipid-lowering and anti-diabetic medication was taken by 52 and 8 patients, respectively. 4 patients were active smokers, 17 were ex-smokers. Left ventricular ejection fraction was mildly reduced (40 – 60%) in 11 patients and severely reduced (<40%) in 18 patients. According to NYHA dyspnea classification, 68, 19, 12 and 0 patients were class I, II, III and IV, respectively. Average FMD was lower in patients with cardiovascular diseases than in healthy adults (men: 4.6 ± 3.2% vs. 6.6 ± 4.0%, women: 4.3 ± 3.5% vs. 7.8 ± 4.3%, *p* < 0.001), whereas there was only little evidence for a difference in L-FMC between groups (men: −0.6 ± 2.8 vs. −0.3 ± 2.0, *p* = 0.30, women: −0.7 ± 3.1 vs. −1.2 ± 3.0, *p* = 0.38). Further measurement data of endothelial function (i.e., shear rate, blood flow, vascular wall thickness) are presented in the Supplementary Table 1.

**TABLE 1:** Sample characteristics.

	Without cardiovascular disease (*N* = 360)		With cardiovascular disease (*N* = 99)	
	Male (*N* = 187)	Female (*N* = 173)	Male (*N* = 71)	Female (*N* = 28)
Age [years]	49.0 ± 18.3	51.2 ± 19.0	68.5 ± 12.5*	72.0 ± 13.4*
$\dot{\text{V}}$O_2_peak [ml/min/kg]	40.5 ± 9.5	32.4 ± 8.0	24.9 ± 8.5*	19.4 ± 5.9*
BMI [kg/m^2^]	24.0 ± 2.4	22.6 ± 2.5	27.3 ± 3.5*	25.5 ± 3.3*
MAP [mmHg]	94.9 ± 8.2	91.1 ± 9.5	97.3 ± 10.8	97.7 ± 10.7*
HbA1c [%]	5.2 ± 0.3	5.2 ± 0.4	5.8 ± 0.6*	5.6 ± 0.4*
Triglycerides [mg/dL]	131.3 ± 71.5	99.8 ± 48.9	125.8 ± 73.1	140.5 ± 88.8*
LDL-cholesterol [mg/dL]	118.2 ± 25.9	123.6 ± 29.2	101.4 ± 31.4*	123.6 ± 31.1
hs-CrP [mg/L]	1.8 ± 4.1	1.9 ± 3.7	3.0 ± 3.8*	2.5 ± 2.0
Creatinine [mg/L]	0.9 ± 0.1	1.3 ± 0.8	1.1 ± 0.4*	0.8 ± 0.2
NT-pro-BNP [pg/mL]	77.9 ± 75.1	128.5 ± 96.1	468.1 ± 667.3*	564.8 ± 829.5*
FMD [%]	6.6 ± 4.0	7.8 ± 4.3	4.6 ± 3.2*	4.3 ± 3.5*
L-FMC [%]	−0.6 ± 2.8	−0.7 ± 3.1	−0.3 ± 2.0	−1.2 ± 3.0
Pre-cuff-infl. diam. [mm]	4.2 ± 0.5	3.4 ± 0.4	4.4 ± 0.6	3.7 ± 0.6*
Resting heart rate [bpm]	57.1 ± 9.2	58.7 ± 8.6	58.4 ± 8.9	61.5 ± 9.8

$\dot{V}$O_2_peak = peak oxygen consumption; BMI = Body mass index; MAP = Mean arterial pressure; HbA1c = glycated hemoglobin; LDL-cholesterol = Low-density lipoprotein-cholesterol; hs-CrP = high-sensitivity C-reactive protein; NT-pro-BNP = n-terminal-pro-brain natriuretic peptide; FMD = flow-mediated vasodilation; L-FMC = low flow-mediated vasoconstriction; Pre-cuff-infl. diam. = pre-cuff-inflation diameter; *significantly different from “without cardiovascular disease” (independent student’s t-test, significance level p = 0.05).

### Association of Endothelial Function With Age

FMD showed a significant negative association with age in the healthy sample without burden of chronic diseases (adjusted R^2^ = 0.27, partial R^2^ = 0.07, *p* < 0.001; Supplementary Table 2) and in patients with cardiovascular diseases (adjusted R^2^ = 0.29, partial R^2^ = 0.05, *p* = 002). L-FMC showed no significant association with age in both samples (Supplementary Table 3).

### Moderating Effects of Cardiorespiratory Fitness on the Decline of Flow-Mediated Vasodilation With Aging

**FIGURE 2:**
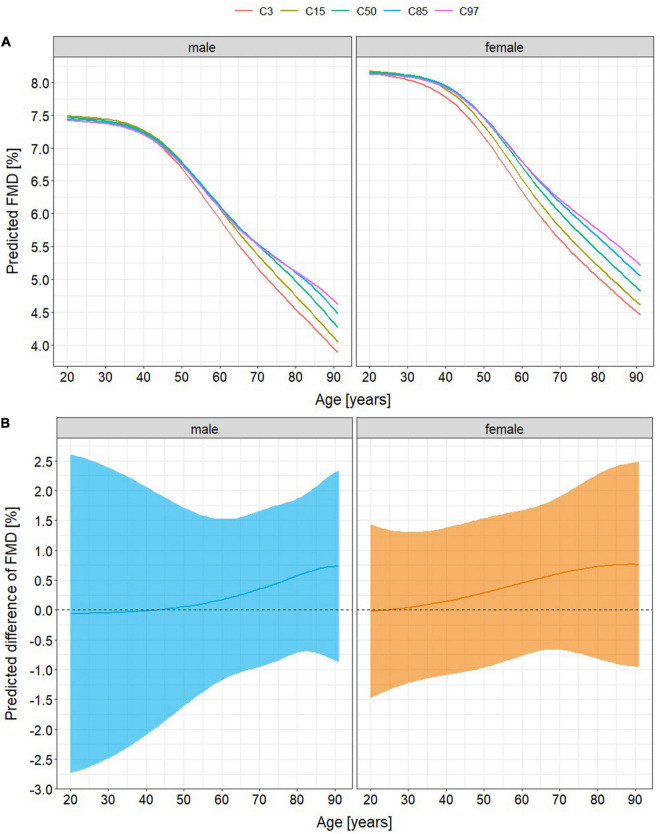
Age-related differences of flow-mediated vasodilation (FMD) in healthy adults **(A)** stratified by percentile of peak oxygen consumption ($\dot{\text{V}}$O_2_peak) and **(B)** predicted difference between FMD in the lowest-fit ($\dot{\text{V}}$O_2_peak ≤3rd percentile) and FMD in the highest fit ($\dot{\text{V}}$O_2_peak ≥97th percentile) individuals. There are no differences associated with cardiorespiratory fitness until the age of 30 (women) and 50 years (men). Afterward, models predict a lower FMD at old age of 7% in very low-fit men (maximum age-related difference of 41% if $\dot{\text{V}}$O_2_peak ≥97th percentile versus 48% if $\dot{\text{V}}$O_2_peak ≤3rd percentile) and of 9% in very low-fit women (36% versus 45%). Absolute mean differences of FMD between $\dot{\text{V}}$O_2_peak percentiles ≤3rd versus ≥97th deviate from 0 in tendency at middle and old age (maximum 0.75%, 95% confidence intervals –0.8 – 2.3% (men) and –1.0 – 2.5% (women)). C3, 15, 50, 85, and 97 = $\dot{\text{V}}$O_2_peak percentiles (3rd, 15th, 50th, 85th, and 97th).

**FIGURE 3:**
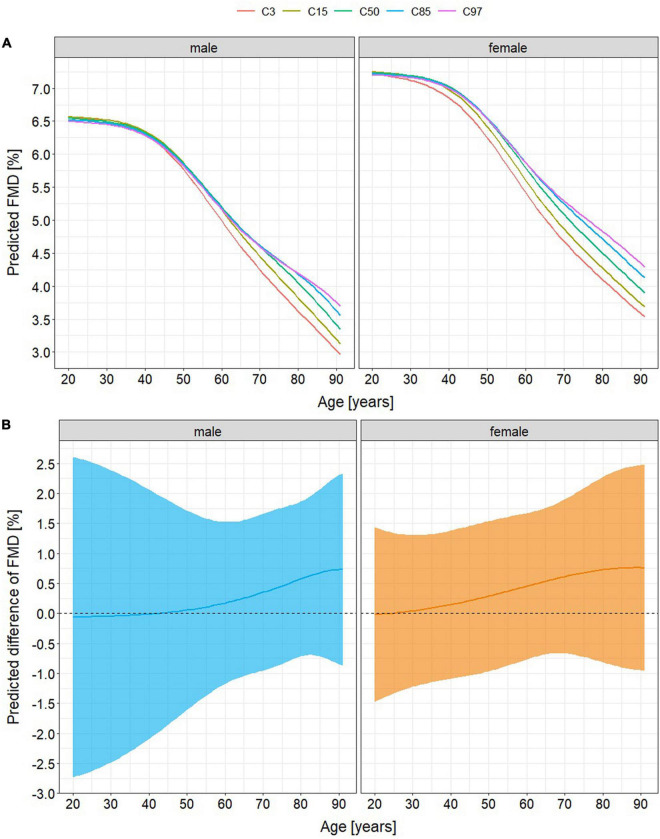
Age-related differences of flow-mediated vasodilation (FMD) in patients with cardiovascular diseases **(A)** stratified by percentile of peak oxygen consumption ($\dot{\text{V}}$O_2_peak) and **(B)** predicted difference between FMD in the lowest-fit ($\dot{\text{V}}$O_2_peak ≤3rd percentile) and FMD in the highest fit ($\dot{\text{V}}$O_2_peak ≥97th percentile) individuals. There are no differences associated with cardiorespiratory fitness until the age of 30 (women) and 50 years (men). Afterward, models predict a lower FMD alt old age of 12% in very low-fit men (maximum age-related difference of 43% if $\dot{\text{V}}$O_2_peak ≥97th percentile versus 55% if $\dot{\text{V}}$O_2_peak ≤3rd percentile) and of 11% in very low-fit women (41% versus 52%). Absolute mean differences of FMD between $\dot{\text{V}}$O_2_peak percentiles ≤3rd versus ≥97th deviate from 0 in tendency at middle and old age (maximum 0.75%, 95% confidence intervals –0.8 – 2.3% (men) and –1.0 – 2.5% (women)). C3, 15, 50, 85, and 97 = $\dot{\text{V}}$O_2_peak percentiles (3rd, 15th, 50th, 85th, and 97th).

There was little evidence that the relationship of FMD with age was moderated by $\dot{\text{V}}$O_2_peak (χ^2^(5) = 5.37, *p* = 0.372, *s* = 1.43). Therefore, models containing no interaction between $\dot{\text{V}}$O_2_peak and age were presented. The model explained 30.3% of the variance of FMD with $\dot{\text{V}}$O_2_peak accounting for 2.8%. It predicted a 41% decline of FMD in very high-fit healthy men ($\dot{\text{V}}$O_2_peak ≥97th percentile) from 20 to 90 years of age compared to 48% in very low-fit healthy men ($\dot{\text{V}}$O_2_peak ≤3rd percentile) and of 36% in very high-fit healthy women compared to 45% in very low-fit healthy women (Figures 2A,B). In patients with cardiovascular diseases, predicted age-related differences of FMD were 43% in very high-fit men compared to 55% in very low-fit men and 41% in very high-fit women compared to 52% in very low-fit women (Figures 3A,B).

## Discussion

Independent of classical cardiovascular risk factors FMD, but not L-FMC, is negatively associated with higher age in both, healthy aging adults and patients with cardiovascular diseases. Additionally, the current study provides some novel insights into the effects of cardiorespiratory fitness on the age-related changes of endothelial function. First, $\dot{\text{V}}$O_2_peak may account only for a small proportion of the age-related change of FMD. Thereby, a constantly low $\dot{\text{V}}$O_2_peak might lead to a 7 (men) – 9% (women) lower FMD at old age in healthy individuals and to a 11 (women) – 12% (men) lower FMD at old age in patients with cardiovascular diseases. Second, this accelerated decline of FMD may become apparent around the age of 30 years in women, thus 20 years earlier than in men.

These study results demonstrate a negative association of FMD with age, but provide little statistical evidence for an influence of $\dot{\text{V}}$O_2_peak on this association. This is in accordance with the results of Montero et al. (14) who reported an age-independent association of $\dot{\text{V}}$O_2_peak with FMD in only 46% of all cross-sectional studies. Furthermore, they also reported that FMD in healthy young adults did not differ in low-fit individuals from those being high-fit, which might be due to generally high adaptive capacities in the vascular walls of the young (15). Assuming that these capacities decline with aging, it seems a logical consequence that the theoretical models in this study suggest relatively lower FMD of 7 – 12% in the lowest-fit old-aged individuals compared to those with the highest $\dot{\text{V}}$O_2_peak (maximum difference of total FMD values was 0.75%). Favorable effects of a high $\dot{\text{V}}$O_2_peak on endothelial function at any age were previously demonstrated (13) emphasizing its importance for a healthy vascular aging independent from other cardiovascular risk factors.

Three potential explanations are proposed for why the statistically small effect of $\dot{\text{V}}$O_2_peak on the negative association of FMD with age might carry a clinical relevance. First, a high cardiorespiratory fitness might exert favorable effects on vascular function that are unrelated to vasodilator function. Therefore, L-FMC was included in the analyses, which was suggested as a complementary biomarker to FMD for the thorough clinical interpretation of endothelial function (18, 19). However, only very little evidence was found for an association of age with L-FMC in this study’s sample, probably due to the large variation of L-FMC values despite strict adherence to current measurement guidelines (30). Furthermore, a previous study comparing L-FMC of the radial with the brachial artery confirmed vasoconstriction upon cuff-occlusion in the radial but not in the brachial artery (39). The authors concluded, that endothelial dysfunction would manifest as attenuated L-FMC in the radial artery but not in the brachial artery. Accordingly, lower vasoconstrictor responsiveness with higher age might rather occur in the smaller resistance arteries instead of the larger ones.

Second, structural remodeling of the vascular wall in terms of thinner thickness with higher $\dot{\text{V}}$O_2_peak was also discussed as an explanation for a partial non-detectability of favorable vascular effects by measurement of FMD and L-FMC (40). However, there was no evidence for an association between brachial arterial wall thickness and $\dot{\text{V}}$O_2_peak in this study’s analyses (Supplementary Table 4) leaving it open for discussion, whether favorable structural adaptations in the vascular walls could mitigate improved FMD and L-FMC (41).

A third explanation can be derived from the assumption, that not maximal shear stress, but rather repeated bouts of increased shear stress induce favorable improvements of endothelial function by exercise (42). Accordingly, light- or moderate intensity exercise that do not induce changes of $\dot{\text{V}}$O_2_peak, might still induce favorable vascular effects (43). At this point, longitudinal studies collecting detailed information about the cumulative load of exercise and physical activity at different intensities are lacking. Furthermore, interventional studies including high-, moderate and low-intensity activities are needed as well, to clarify how different modes of exercise effectuate long-term endothelial function independent of $\dot{\text{V}}$O_2_peak.

### Clinical Considerations

Although statistically non-significant, the predicted 7 – 9% reduction of FMD with higher age in association with a constantly low $\dot{\text{V}}$O_2_peak in the healthy sample should not be ignored for their potential clinical relevance on an individual level. Notably, a 1% higher FMD may lead to a 13% reduction of cardiovascular morbidity (44, 45) and, thus, might reduce the risk of cardiovascular morbidity considerably, regardless of sex, age or other cardiovascular risk factors (44, 45).

In the older patients with cardiovascular diseases, FMD was 11 (women) – 12% (men) lower in the lowest $\dot{\text{V}}$O_2_peak percentile compared to the highest, which equals an absolute difference of 0.8%. This is similar to previously observed effects of exercise training in heart failure patients increasing FMD by 1.08% (11), which, again seems clearly relevant, in terms of cardiovascular morbidity (44, 45). This is supported by further observations. For example, one study on Japanese individuals with coronary artery disease observed 50% lower rates of cardiovascular events with every 3.1% total improvement of FMD (46). Furthermore, a reduced FMD was previously associated with higher rates of in-stent restenosis (47) and occurrence of postoperative cardiovascular sequelae (48) in patients with coronary artery disease.

Differences in FMD stratified by $\dot{\text{V}}$O_2_peak percentile became visible at the age of 30 years in women, thus 20 years earlier than in men. This seems counter-intuitive, as previous evidence suggests a 10 year earlier decline of endothelial function in men than in women indicating their higher risk for early cardiovascular morbidity and mortality (49). However, this study’s observations support the assumption, that $\dot{\text{V}}$O_2_peak-improving exercise during young adulthood might exert higher favorable effects on vasodilator responsiveness in women, than in men (13).

### Methodological Considerations

The quality of phenotyping with direct state-of-the art measurement of cardiorespiratory fitness and endothelial function as well as the large and well-phenotyped samples representing the healthy population and a population with high cardiovascular disease burden are major strengths of this study.

Importantly, results from the regression analyses are visualized in Figures 2, 3 but not based on visual inspection of these graphs. Furthermore, they are not descriptive and do not provide clinical cut-offs for FMD with high or low $\dot{\text{V}}$O_2_peak. For this, longitudinal studies with multiple measures are needed. Due to their cross-sectional nature, the models do not allow for the conclusion, that people with a high $\dot{\text{V}}$O_2_peak throughout their adult life will show considerably better FMD than those with low cardiorespiratory fitness, especially as the process of aging does not follow a linear course, thus ultimately requesting longitudinal studies to be assessed. Yet, a person will most likely have a better individual FMD, if a high $\dot{\text{V}}$O_2_peak is maintained into old age. However, the current results give valuable insights into the relationship between vascular aging and cardiorespiratory fitness, supporting previous claims that direct effects of exercise on vascular function and structure might constitute a relevant part of the beneficial effects of exercise in humans independent of classical cardiovascular risk factors (6, 50).

## Conclusion and Perspectives

Flow-mediated vasodilation is negatively associated with age in a healthy population as well as in patients with cardiovascular diseases, whereas L-FMC is not. $\dot{\text{V}}$O_2_peak explains only a small proportion of the age-related change of FMD independent of traditional cardiovascular risk factors. This small statistical effect might account for up to 7–12% lower values at old age in low-fit healthy adults and patients with cardiovascular diseases compared to those with higher lifelong $\dot{\text{V}}$O_2_peak. Especially in the patients, this might be clinically relevant in terms of increased morbidity and mortality, emphasizing the need to aim for a lifelong preservation of cardiorespiratory fitness and endothelial function. As a consequence, clinicians should promote the implementation of regular intensive exercise at any age in both, healthy adults as well as patients with cardiovascular diseases. Scientists should conduct long-term observational studies as well as training interventions to analyze $\dot{\text{V}}$O_2_peak-independent benefits of cumulative exercise loads and of different exercise intensities on endothelial function.

## Data Availability Statement

The original contributions presented in the study are included in the article/Supplementary Material, further inquiries can be directed to the corresponding authors.

## Ethics Statement

The studies involving human participants were reviewed and approved by Ethikkommission Nordwestschweiz, Switzerland. The patients/participants provided their written informed consent to participate in this study.

## Author Contributions

KK wrote the manuscript, conducted data analysis and participated in the conception, and conduction of the study. JW participated in the conception and conduction of the study and extensively engaged in preparation, and revision of the manuscript drafts. DI participated in the conception of the study, data analysis, and revision of the manuscript drafts. RK, GN, CK, and JC participated in the conduction of the study and extensively engaged in preparation, and revision of the manuscript drafts. TH and AS-T participated in the conception of the study, supervised the conduction and data analysis and extensively engaged in preparation, and revision of the manuscript drafts. All authors contributed to the article and approved the submitted version.

## Conflict of Interest

The authors declare that the research was conducted in the absence of any commercial or financial relationships that could be construed as a potential conflict of interest.

## Publisher’s Note

All claims expressed in this article are solely those of the authors and do not necessarily represent those of their affiliated organizations, or those of the publisher, the editors and the reviewers. Any product that may be evaluated in this article, or claim that may be made by its manufacturer, is not guaranteed or endorsed by the publisher.

## Abbreviations

FMD: Flow-mediated dilation
L-FMC: Low flow-mediated constriction
$\dot{\text{V}}$O_2_peak: Peak oxygen consumption.
