# Supplementary material for: Cardiorespiratory Fitness and Endothelial Function in Aging Healthy Subjects and Patients With Cardiovascular Disease
Source: Front Cardiovasc Med. 2022 Apr 28;9:870847. doi: 10.3389/fcvm.2022.870847 (PMC9095821; doi:10.3389/fcvm.2022.870847)
Supplement: Supplementary file 1 [file Data_Sheet_1.PDF]

Supplementary materials

Table S1: Raw data of measurement of endothelial function.

	Without cardiovascular disease (N = 360)		With cardiovascular disease (N = 99)	
	Male (N = 187)	Female (N = 173)	Male (N = 71)	Female (N = 28)
Pre-cuff-inflation				
Diameter [mm]	4.24 ± 0.50	3.38 ± 0.40	4.38 ± 0.63	3.67 ± 0.57*
Blood flow velocity [cm/s]	4.30 ± 2.57	3.12 ± 1.92	3.93 ± 3.03	2.73 ± 1.67
Pre-cuff-deflation				
Diameter [mm]	4.21 ± 0.50	3.36 ± 0.40	4.37 ± 0.64*	3.62 ± 0.57*
Post-cuff-deflation				
Peak diameter [mm]	4.51 ± 0.49	3.64 ± 0.40	4.58 ± 0.62	3.82 ± 0.56*
Peak blood flow velocity [cm/s]	31.27 ± 15.78	22.51 ± 11.36	22.86 ± 13.49*	16.70 ± 10.40*
Peak sheart rate	603.20 ± 317.10	526.70 ± 280.47	441.26± 238.65*	385.35 ± 219.84*
time of peak diameter [s]	57.44 ± 16.47	53.20 ± 16.50	57.43 ± 18.24	55.84 ± 17.83
time of peak shear rate [s]	9.30 ± 4.64	9.83 ± 5.58	9.00 ± 5.08	9.79 ± 6.17
time between peak diameter and peak shear rate [s]	48.14 ± 16.45	43.37 ± 17.14	48.55 ± 19.20	46.05 ± 16.81

Data presented as mean ± standard deviation; * = significantly different from "without cardiovasculardisease" (independent student's t-test, significance level $p = 0.05$).

Table S2: Linear regression model of the association between age and FMD in the healthy sample (a) and in cardiovascular patients (b).

(a)

Dependent variable: FMD; Model fit: adjusted $R^2 = 0.2654$	Df	partial R^2	F	*p*
Age	3	0.06879474	13.3576	<0.0001*
Heart rate	3	0.01358515	4.0788	0.007239*
Mean arterial pressure	3	0.01958925	3.0910	0.027187*
Diameter	3	0.14455550	18.7632	<0.0001*
Sex	1	0.01891217	7.1564	0.007825*

*significance level $p = 0.05$

(b)

Dependent variable: FMD; Model fit: adjusted $R^2 = 0.2935$	Df	partial R^2	F	*p*
Age	3	0.04747345	3.3960	0.02151*
Heart rate	3	0.03238808	0.9154	0.43709
Mean arterial pressure	3	0.04747080	1.7166	0.16968
Diameter	3	0.13442645	3.3725	0.02214*
Sex	1	0.03173637	7.1090	0.00918*

*significance level $p = 0.05$

Table S3: Linear regression model of the association between age and L-FMC in the healthy sample (a) and in cardiovascular patients (b).

(a)

Dependent variable: L-FMC; Model fit: adjusted R^2 = 0.0474				
	Df	partial R^2	F	*p*
Age	3	0.014556343	1.9907	0.1151
Heart rate	3	0.014556343	0.5865	0.6242
Mean arterial pressure	3	0.006614096	0.2428	0.8665
Diameter	3	0.016005182	1.7311	0.1603
Sex	1	0.004681685	3.5663	0.0598

*significance level p=0.05

(b)

Dependent variable: L-FMC; Model fit: adjusted R^2 = 0.0864				
	Df	partial R^2	F	*p*
Age	3	0.005925127	0.3154	0.8142
Heart rate	3	0.018127282	0.4817	0.6958
Mean arterial pressure	3	0.031203873	0.9208	0.4344
Diameter	3	0.005169272	0.3442	0.7934
Sex	1	0.026007527	1.8552	0.1768

*significance level p = 0.05

Table S4: Linear regression model of the association between V̇O2peak and brachial arterial wall thickness (baWT).

Dependent variable: baWT; Model fit: adjusted R^2 = 0.1454				
	Df	partial R^2	F	*p*
V̇O2peak	3	0.021009173	0.0809	0.970380
Age	3	0.074775341	5.4318	0.001155*
Heart rate	3	0.009463265	1.2925	0.276733
Mean arterial pressure	3	0.007772154	0.5142	0.672713
Diameter	3	0.023854290	2.2114	0.086445
Sex	1	0.003582339	0.0962	0.756576
CVD	1	0.004946248	0.2266	0.634335

*significance level p = 0.05
